# Evaluation of Educational Materials on Colorectal Cancer Screening in Appalachian Kentucky

**Published:** 2006-03-15

**Authors:** RaeAnne E Davis, Debra K Armstrong, Jennifer Redmond, Mark Dignan, Gretchen R Norling

**Affiliations:** Tobacco Prevention & Cessation Program, Chronic Disease Prevention and Control Branch, Kentucky Department for Public Health; At the time this study was conducted, Ms Davis was affiliated with the Kentucky Cancer Program, University of Kentucky Markey Cancer Control Program, Lexington, Ky; Kentucky Cancer Program; Kentucky Cancer Program; Prevention Research Center, University of Kentucky Markey Cancer Control Program, Lexington, Ky; Cancer Information Service Mid-Atlantic Region, Mary Babb Randolph Cancer Center, Robert C. Byrd Health Sciences Center of West Virginia University, Morgantown, WVa

## Abstract

**Introduction:**

Despite the availability of preventive screening for colorectal cancer, compliance with screening recommendations in Appalachian Kentucky is low. Although there are various cancer education materials available, none focus on Appalachian populations and few on low-literacy populations. The purpose of this study was to assess the type of information needed in written educational materials about colorectal cancer for Appalachian populations in Kentucky.

**Methods:**

Seven focus groups were held in two Appalachian regions of Kentucky. Thirty-four members of the community participated in four focus groups held for the general public, and 15 staff members of primary care physicians' offices participated in three focus groups. One facilitator led all seven focus groups using a moderator's guide. Participants were asked to review and rank two fact sheets and two brochures about colorectal cancer according to perceived effectiveness.

**Results:**

There was consensus between the general public focus groups and physician office staff focus groups about the ranking of materials. All groups preferred the Centers for Disease Control and Prevention's *Screen for Life: National Colorectal Cancer Action Campaign* fact sheet and brochure to the other materials. They indicated that factors such as print size, inclusion of diagrams, and clear and simple presentation of the information were important and made the materials easier to use and understand. A consensus was also reached among groups on the relative importance of types of information that should be provided in the materials.

**Conclusion:**

The use of educational materials to communicate messages about cancer screening is important in increasing awareness and providing valuable health information. Members of the Appalachian community and staff members of physicians' offices preferred and recommended use of *Screen for Life* materials for low-literacy and Appalachian populations over other educational materials.

## Introduction

According to the Centers for Disease Control and Prevention (CDC) and the Kentucky Department for Public Health, colorectal cancer is the second most common cause of cancer-related deaths in both the United States and Kentucky (1,2). The American Cancer Society estimated that in 2005, 2350 new cases would be diagnosed, and 910 deaths would occur in Kentucky (3). Studies have indicated that screening tests are effective in preventing colorectal cancer and detecting it early. Identification through screening and removal of precancerous polyps can prevent colorectal cancer. Screening tests can also detect colorectal cancer at early stages of disease, allowing for earlier treatment and increased survival rates (4-8). Clinical studies show that only 37% of all colorectal cancers are diagnosed in their earliest stages, when the cancers are still localized and survival rates are highest (8).

The U.S. Preventive Services Task Force (USPSTF) strongly recommends routine screening for colorectal cancer beginning at age 50 years for both men and women of average risk. USPSTF recommends an annual fecal occult blood test, a flexible sigmoidoscopy or double barium enema every 5 years, and a colonoscopy every 10 years (9). Despite the availability of efficacious screening, compliance with screening recommendations for this disease is low. According to CDC's 2002 Behavioral Risk Factor Surveillance System (BRFSS), 43.9% of Kentucky respondents aged 50 years or older indicated ever having had a sigmoidoscopy or colonoscopy examination compared with 48.1% of respondents in the United States (10).

### Factors related to cancer screening

The Task Force on Community Preventive Services conducted a review of population-based interventions designed to promote informed decision making about cancer screening. It found that use of information in brochures and on Web sites helped individuals learn about the types of screening available and make decisions about when to be screened for cancer (11). However, low literacy can negatively affect an individual's ability to process and understand health information and concepts, such as screening and early detection (12,13). Davis et al have highlighted the importance of literacy in understanding educational messages related to cancer screening (14), and Doak et al have highlighted the importance of pilot testing educational materials to determine their effectiveness in conveying information (15). Low literacy is often associated with limited education and low income (14). Davis et al reported a lower rate of colorectal cancer screening among 2002 BRFSS respondents who had lower income and educational attainment (16). This relationship is also reflected in 2002 BRFSS colorectal cancer screening rates reported for Kentucky.

### Study setting

Of the 120 counties in Kentucky, 51 (42%) are designated as Appalachian by the Appalachian Regional Commission (17). The National Cancer Institute's Center to Reduce Cancer Health Disparities has recognized Appalachians as a special population because of their health and socioeconomic disparities compared with the general U.S. population. Appalachian Kentucky has a largely rural population. Systemic factors such as lack of public transportation, fewer community services, and a shortage of health care providers make the use of written materials to communicate health care messages essential (18,19). Education, income, and access to health care have been found to be factors that influence the success of educational programs (20). 

As shown in Table 1, 2000 U.S. census data indicate that Appalachian Kentucky has a higher poverty rate and a lower rate of adults who have a high school diploma than the United States as a whole (21). Table 2 shows the colorectal cancer screening rates among adults aged 50 years and older for Appalachian Kentucky, non-Appalachian Kentucky, and the state as a whole. Only 38.1% of residents in Appalachian Kentucky reported ever having a sigmoidoscopy or colonoscopy, compared with 46.2% of residents in non-Appalachian areas of Kentucky (22).

A variety of educational materials on colorectal cancer is available; however, none has been developed specifically for Appalachian populations. The purpose of this study was to assess the type of information needed in written educational materials about colorectal cancer for Appalachian populations in Kentucky.

## Methods

The Kentucky Cancer Program (KCP) at the University of Kentucky Markey Cancer Center and the Appalachia Cancer Network jointly sponsored a pilot study in two Appalachian Kentucky communities. The study used focus groups to assess the effectiveness of existing colorectal cancer screening educational materials as perceived by the general public and by staff of primary care physicians' offices. The focus groups were structured using guidelines established by Morgan and Krueger (23). Physician office staff were included in the study because they have extensive experience with patients and know their need for educational materials, their reading levels, and the barriers they face in obtaining screening. The medical office staff regularly interact with patients and are in a position to provide appropriate educational materials. In addition, staff members often have the responsibility for ordering, stocking, and recommending health communication materials.

KCP partnered with the University of Kentucky Prevention Research Center and Department of Communication to conduct the focus groups. The purpose of the focus groups was to 1) rank selected colorectal cancer screening educational materials according to effectiveness perceived by the general public and primary care physicians' office staff and 2) determine if there are any differences between the general public and primary care office staff in perceived effectiveness of the materials.

### Focus group participants

KCP regional cancer control specialists recruited the participants with the help of key informants and regional cancer advisory councils, as well as through newspaper advertisements and flyers. All participants were volunteers from Appalachian communities in Kentucky. Eligibility criteria for the general public focus groups included living in Appalachian Kentucky, being aged 50 or older, and not having been previously diagnosed with colorectal cancer. The criterion for the office staff focus groups was being employed with a primary care physician's practice in an Appalachian community.

Four focus groups were conducted with the general public, and three focus groups were conducted with staff from two primary care physicians' offices. The research protocol was reviewed and approved by the University of Kentucky Institutional Review Board. Participants reviewed and signed consent forms and completed a brief demographic survey. Light refreshments were provided at the focus groups, and participants were paid $30 each for their participation.

### Materials reviewed

Participants reviewed two fact sheets and two brochures. The fact sheets were from CDC's *Screen for Life: National Colorectal Cancer Action Campaign (Screen for Life)* (24) and the Cancer Research and Prevention Foundation (25). The brochures were from *Screen for Life* (26) and the American Cancer Society (27). These materials are currently being used throughout Kentucky by cancer control specialists, health departments, and health educators to increase awareness about colorectal cancer and the importance of screening to prevent colorectal cancer.

### Moderator's guide

A moderator's guide was developed with assistance from the University of Kentucky's Department of Communication. The guide included a set of questions that were used consistently in all focus groups. The questions centered on content, aesthetic qualities, and usability. Transcripts of the interviews were prepared and analyzed for content using standard methods of qualitative analysis. A researcher from the Department of Communication was the moderator for all of the focus groups. The moderator met with KCP project staff before conducting the focus groups to review the questions and research protocol. All focus groups were audiotaped. The moderator analyzed the focus group transcripts using standard methods of qualitative analysis and submitted a project report. Project staff reviewed the final report.

## Results

### Focus group participants

Thirty-four participants from the general public aged 50 years and older who had never been diagnosed with colorectal cancer participated in one of four 90-minute focus groups conducted in Ashland and Somerset, Ky. All participants were white; the majority (79%) were female, and ages ranged from 50 to 85 years. The education level ranged from less than a high school diploma to a master's degree; 47% had less than or equal to a high school diploma or a general equivalency diploma or had completed a technical school program. Among the participants, 53% did not know anyone who had been diagnosed with colorectal cancer (a family member or friend).

Fifteen staff members from primary care physicians' offices participated in the office staff focus groups; two of these focus groups were held in Ashland and one in Somerset, Ky. All of the participants were white women ranging in age from 25 to 55 years. The education level for the primary care office staff ranged from a high school diploma to a college degree. Forty-seven percent had a technical or associate's degree, and the majority of participants (73%) knew someone who had been diagnosed with colorectal cancer.

### Overall ranking of materials

All groups preferred the *Screen for Life* fact sheet and brochure to the other organizations' materials. Their stated reasons for preferring the *Screen for Life* materials were that they are clear and simple, contain a diagram of the colon, have larger print, use color, and use less intimidating language. These factors helped to make the materials more "user friendly." There was general agreement that the materials had similar types and quantity of information but that the information was presented differently. The brochure was identified as being easier to handle than the fact sheet because it was more portable and could easily fit into a purse or pocket.

There was also agreement between the general public and physician office staff that both the *Screen for Life* fact sheet and brochure were the better choices for low-literacy audiences. The use of pictures or diagrams and easily understood language were considered important in selecting the *Screen for Life* materials. Focus group participants said that using language that was "too wordy" and "intimidating" would discourage many people from picking up or reading educational materials.

### Ranking the importance of information

Participants in the general public and office staff focus groups were asked to rank the importance of information contained in the educational materials on a scale of 1 to 10, with 1 being "not important" and 10 being "very important." Some items participants were asked to rank for importance included 1) risk factors and identifying individuals at risk, 2) list of symptoms, 3) description of screening tests, 4) price estimates for screening tests, 5) diagrams of colon and rectum, and 6) insurance coverage for each test. There was consensus between the general public and office staff groups about the ranking of information perceived to be important. The following five information items were identified as most important to include in educational materials on colorectal cancer: 1) colorectal cancer is preventable; 2) screening saves lives; 3) diagram of the colon; 4) risk factors, symptoms, and warning signs; and 5) screening tests.

Nearly all focus group participants felt that it was very important to state in the materials that colorectal cancer is preventable and that screening saves lives. It was recommended that this information be prominently displayed. One of the participants said:

I have many family members who have died of different forms of cancer, and I think just the sound of the word scares people, but when you put the word *preventable* in there, I think that immediately gets the attention of someone. I can maybe prevent myself from going through what I saw someone else go through.

Having a diagram of the colon was considered important, and most of the participants rated this factor as a 10. Listing who is at risk (including age) and what the risk factors are, along with the symptoms and different screening tests, were also important. The majority of participants rated these elements at an 8 or above in importance.

Most participants in all focus groups agreed that knowing the details about the cost and insurance coverage for each test was not very important. It was suggested that including a general statement that the tests are usually covered by insurance would suffice. Several participants pointed out that knowing the actual cost may discourage people from having the test, even if financial assistance was available.

Opinions among participants were mixed about whether to include a detailed description and list of advantages and disadvantages for each type of screening test. The differences of opinion appeared to be based on an individual's information-seeking behavior. Participants who identified themselves as preferring additional information rated detailed information as high in importance. One participant indicated, "I want details; I have to have every one of them." In contrast, participants who were not interested in additional information indicated that detailed information was not as important and suggested that it might actually discourage people from being screened. This viewpoint was reflected in one participant's comment: "I don't care what you do to me as long as I don't know it. . . . That's really a personal thing with me. . . . Just get it done, fix me, and wake me up."

### Suggestions for additional information

The focus group participants were asked about important information that was missing in the materials. Participants offered suggestions about information that would be valuable to include if changes could be made. One participant suggested including screening test timetables, especially if the frequency of testing is different for low-risk or high-risk individuals. It was noted that the materials do not mention where screening tests are performed. Some participants mentioned that there is no discussion of alternative or complementary medicine, such as vitamins and supplements, or other factors that might make a difference.

It was also pointed out that although "having an active lifestyle" is an often-used phrase, it is rarely defined. As one participant put it, "What my mother thinks is an active lifestyle as compared to what I think is an active lifestyle are two different things." Another participant commented that it should be made clear whether sedation is used for each test. In addition, it was suggested that more information about prevention should be in the materials, such as eating more fruits and vegetables, not smoking, and eating less red meat.

## Discussion

Colorectal cancer is the second leading cause of cancer-related deaths in the United States and Kentucky. Screening rates are low, despite research supporting the efficacy of screening in preventing colorectal cancer for people of average risk aged 50 years and older. The use of written materials to communicate screening messages is an important method of increasing awareness and providing health information, especially in rural areas characterized by limited access to health care services and shortages of health professionals.

### Strengths

This study focused on preventive screening; the exclusion criteria helped control general public perceptions and viewpoints that could have been influenced by a personal experience with diagnostic screening or colorectal cancer. Because the study was conducted in Appalachia and the focus group participants had varying levels of education, we determined that the study population was a good representation of the target population for whom the educational materials were being reviewed.

### Limitations

The number of participants in this descriptive study was small; however, we consider the participants to be representative of the population being targeted (i.e., residents of Appalachian communities). The study did not represent all regions of Appalachia, and it did not address sex, urban and rural, or ethnic and racial differences. Including a larger sample of people across the Appalachian region would have provided a better representation of responses. Another limitation was the number of colorectal cancer educational materials reviewed. There were other materials available that were not reviewed because of time constraints.

### Recommendations

Members of the general public and staff from primary care physicians' offices in two Appalachian communities preferred the *Screen for Life* educational materials to the other materials they reviewed. *Screen for Life* materials were also identified as being the most appropriate for low-literacy populations. The focus groups' responses support findings by Beeker et al (28) and Jorgensen et al (29) about the importance of messages that emphasize that colorectal cancer is preventable and that screening saves lives. These messages were perceived to be so important that many participants across all focus groups said this should be the first thing that people see displayed on the materials. 

The majority of participants identified a preference for the brochure format and educational materials that are colorful, use larger print, use simple words, and have diagrams or pictures. The preference for easy-to-read materials is also highlighted by Weiss and Coyne (30). They recommend that essential written materials be prepared at a fifth-grade reading level or lower. Simple and clear messages improve comprehension and understanding. In addition, augmenting written materials with pictures and diagrams can increase effectiveness with low-literacy populations.

The general public and primary care physicians' office staff believed it was important to include information about risk factors and symptoms. This view may indicate a need for increased educational efforts that emphasize that risk increases with age, that all men and women aged 50 and older need to be screened, and that colorectal cancer can develop without specific symptoms.

There were mixed opinions within the groups about the importance of including detailed information on screening tests and symptoms. Information-seeking styles affected the amount of information that was desired. When educating large groups, presenters must recognize that educational materials may not be appropriate for everyone, and the level of information desired may vary. Using basic educational materials for presentations, complemented by more detailed information for individuals who are information seekers, may be one approach to addressing the differences in the level of information desired.

This research study highlights the importance of developing educational materials that are clear and simple to understand for low-literacy populations and demonstrates the importance of pilot testing the usability of educational materials with target populations.

## Figures and Tables

**Table 1:** Poverty Rate and Percentage of Population Aged 25 Years and Older With a High School Diploma in Appalachian Kentucky, Kentucky, and the United States^a^

**Region**	**Poverty Rate (1999), %**	**High School Diploma (2000), % **
Appalachian Kentucky	24.4	62.6
Kentucky	15.8	74.1
United States	12.4	80.4

a Source: U.S. Census Bureau (21).

**Table 2:** Colorectal Cancer Screening Among Individuals Aged 50 Years and Older in Appalachian Kentucky, Non-Appalachian Kentucky, and Kentucky, Behavioral Risk Factor Surveillance System, 2002^a^

**Region**	**Ever Had Sigmoidoscopy or Colonoscopy**	

	**Yes% (95% Confidence Interval)**	**No% (95% Confidence Interval)**
Appalachian Kentucky	38.1 (34.6-41.7)	61.9 (58.3-65.4)
Non-Appalachian Kentucky	46.2 (43.1-49.3)	53.8 (50.7-56.9)
Kentucky	43.9 (41.4-46.3)	56.2 (53.7-58.6)

a Source: Kentucky Department for Public Health and Centers for Disease Control and Prevention (22)
